# Association between bee allergy and gastric inflammation: A retrospective study of *Helicobacter pylori* and mucosal findings

**DOI:** 10.1097/MD.0000000000046332

**Published:** 2025-12-19

**Authors:** Berkan Acar, Ali Muhtaroğlu, Kubilay İşsever, Ersin Kuloğlu, Sefer Aslan, Ahmet Cumhur Dülger

**Affiliations:** a Department of General Surgery, Giresun University Faculty of Medicine, Giresun, Turkey.

**Keywords:** bee allergy, dyspepsia, endoscopic biopsy, gastric inflammation, *Helicobacter pylori*

## Abstract

This retrospective and exploratory study aimed to examine the potential association between bee allergy and increased rates of chronic gastric inflammation and *Helicobacter pylori* positivity in patients presenting with dyspeptic symptoms. Medical records of patients with dyspeptic symptoms and self-reported bee allergy who underwent upper gastrointestinal endoscopy at a tertiary care hospital were retrospectively reviewed. Twenty-nine patients with bee allergy were compared with a control group of 29 patients matched by age, gender, and gastrointestinal complaints. Antral biopsy samples were evaluated histopathologically for chronic inflammation, neutrophil activity, *H pylori* presence, atrophy, metaplasia, and dysplasia. Laboratory parameters, including complete blood count, electrolytes, biochemical values, and coagulation profiles, were also analyzed. Patients with bee allergy exhibited significantly higher rates of chronic gastric inflammation, neutrophil activity, and *H pylori* positivity (*P* < .05). No significant differences were observed in hematological, biochemical, or coagulation markers, except for sodium and potassium levels, which were elevated in the bee allergy group (*P* < .05). Path analysis revealed that bee allergy was positively associated with chronic inflammation (β = 0.41, *P* < .05) and *H pylori* positivity (β = 0.38, *P* < .05). The findings of this exploratory study indicate a possible association between bee allergy and increased gastric mucosal inflammation and *H pylori* prevalence. Additionally, electrolyte differences observed in the bee allergy group may reflect broader systemic changes. These preliminary results warrant further investigation through prospective and mechanistic studies.

## 1. Introduction

Allergic reactions are defined as hypersensitivity responses triggered by the immune system following exposure to specific allergens. Among these, bee venom allergy is of particular clinical importance due to the potential of the allergen to cause systemic effects, ranging from localized swelling to life-threatening anaphylaxis.^[1]^ It has been demonstrated that bee venom contains immunogenic peptides and enzymes, which have the capacity to provoke robust immunoglobulin E mediated immune responses, particularly in individuals who have already been sensitized and following repeated stings.^[2]^

Whilst the skin and respiratory systems are well-known targets in systemic allergic reactions, increasing evidence suggests that the gastrointestinal system may also be involved.^[3]^ A range of symptoms, including nausea, abdominal pain, vomiting and diarrhea, have been reported in cases of Hymenoptera sting reactions, suggesting a potential role for the gut in this context. It is hypothesized that these responses are the result of immune-mediated mechanisms, including mast cell degranulation and the release of inflammatory mediators that may compromise mucosal integrity.^[4,5]^

The gastric mucosa, with its dense network of neurohumoral and immune cells, is particularly susceptible to systemic immunological influences. It is hypothesized that chronic allergic inflammation may increase vulnerability of the gastric lining to microbial colonization, especially by *Helicobacter pylori*, a well-established pathogen associated with gastritis, ulcers, and gastric cancer. Recent studies have suggested that immune dysregulation associated with allergic disorders may influence the local inflammatory milieu and epithelial barrier function in the stomach, thereby potentially altering host–microbial interactions.^[6,7]^

Despite these theoretical connections, there is a lack of empirical research investigating the relationship between bee allergy and histopathological or biochemical changes in the gastrointestinal system. The potential repercussions on inflammatory markers, neutrophilic activity, *H pylori* colonization, and metabolic indicators such as electrolyte balance, however, remain to be fully explored.

This retrospective and exploratory study was conducted to evaluate whether individuals with a history of bee allergy exhibit distinct changes to their gastric mucosa and laboratory findings compared to non-allergic individuals with similar dyspeptic symptoms. The present study aims to clarify potential immunological links between bee allergy and gastrointestinal pathology by examining histopathological features and biochemical parameters.

## 2. Materials and methods

The study protocol was approved by the local ethics committee of our hospital on June 11, 2025 with the decision numbered June 11, 2025/05. This study was conducted in accordance with the ethical principles outlined in the Declaration of Helsinki and was approved by the Institutional Review Board at our hospital. Given the retrospective nature of the study, the requirement for informed consent was waived by the Institutional Review Board.

This retrospective comparative study was conducted at our tertiary care hospital, where medical records of patients presenting with dyspeptic symptoms who attended general surgery, internal medicine, and gastroenterology outpatient clinics between September 2021 and December 2024 were reviewed. Patients were included in the study based on the following criteria: the presence of dyspeptic symptoms, the undergoing of upper gastrointestinal endoscopy, and the availability of histopathological biopsy results. The primary distinguishing criterion was the self-reported presence or absence of bee allergy. The bee allergy was defined by patients’ statements indicating a prior bee sting associated with the immediate onset of allergic symptoms (e.g., swelling, urticaria, respiratory distress). According to this criterion, patients were divided into 2 distinct groups. Group 1 (bee allergy group) comprised 29 patients who reported a history of bee allergy. Group 2 (control group) consisted of 29 patients who were matched for age, gender, and clinical presentation but did not have a history of bee allergy.

Antral biopsies were obtained during upper gastrointestinal endoscopy and evaluated by a specialist pathologist who was unaware of the patients’ allergy status. The assessment specifically included evaluation for chronic inflammation, neutrophil activity, *H pylori* positivity, intestinal metaplasia, mucosal atrophy, and dysplasia. The investigation was conducted in a laboratory setting. The patient records provided laboratory data including leukocytes, hemoglobin, hematocrit, mean corpuscular volume, platelet count, lymphocyte and neutrophil counts, serum glucose, urea, creatinine, and alanine aminotransferase, aspartate aminotransferase, albumin, electrolytes (sodium, potassium, calcium), C-reactive protein, ferritin, thyroid-stimulating hormone, prothrombin time, international normalized ratio, and activated partial thromboplastin time.

Endoscopy was performed by gastroenterologists, with the mucosal changes evaluated using high-resolution electronic endoscopy equipment (Fujifilm EPX3500 HD series).

### 2.1. Statistical analysis

Data analysis was performed using the IBM Statistical Package for the Social Sciences (SPSS) 26.0 (IBM Corp., Armonk) statistics software package. The normality of numerical variables in patients with and without bee allergy was determined by examining skewness and kurtosis values. Normally distributed data are denoted by the letter *t*, and non-normally distributed data are denoted by the letter *z*. The reference values for the normal distribution are ± 1.96. The Chi-square test was used to compare the gender, clinical, and demographic findings of patients with and without bee allergies. The Spearman correlation test was used to examine the relationships between bee allergies and demographic, clinical, and laboratory values. The correlation coefficient was evaluated as low (0.00–0.30), moderate (0.30–0.70), and high (0.70–1.00). Path analysis was performed to evaluate the effect of bee allergy on various findings, and the AMOS 24 programme was used for this analysis. Significance levels of .05 and .01 were considered in all analyses.

## 3. Results

The demographic comparison between groups showed no significant differences. Both the bee allergy group (n = 29) and the control group (n = 29) demonstrated similar gender distribution (*P* = .793). Mean ages were comparable across both groups, with no statistically significant difference observed (*P* = .794). Table 1 shows a comparison of gender and age data between groups.

**Table 1:** Comparison of gender and age data between groups.

Variables		Bee allergy group (n: 29)		Control group (n: 29)		*P*
		Number	%	Number	%	
Gender	Female	13	44.8	15	51.7	.793
	Male	16	55.2	14	48.3	
		Med.±SD Med. (Min.–Max.)		Med.±SD Med. (Min.–Max.)		
Age†		53.48 ± 15.45 52 (21–89)		54.55 ± 15.53 57 (25–91)		.794

𝜒^2^: Chi-square test (categorical data).

Max = maximum, Med = median, Min = minimum, SD = standard deviation.

† Independent sample *t* test.

Histopathological evaluation revealed significant differences between groups. Specifically, chronic gastric inflammation was notably more frequent in patients reporting bee allergy compared to controls (*P* = .008). Likewise, increased neutrophil activity was significantly associated with the bee allergy group (*P* = .010). *H pylori* positivity was also significantly higher in the bee allergy group compared to the control group (*P* = .002). No statistically significant differences were found regarding the prevalence of intestinal metaplasia, atrophy, or dysplasia between the 2 groups (*P* = .666, *P* = 1.000). Table 2 shows a comparison of the histopathological results of gastric biopsies between the 2 groups.

**Table 2:** Comparison of the histopathological results of gastric biopsies between the 2 groups.

Variables		Bee allergy group (n: 29)		Control group (n: 29)		*P*
		Number	%	Number	%	
Chronic inflammation	None	0	0.0	8	27.6	**.008** **
	Mild	8	27.6	8	27.6	
	Moderate	13	44.8	11	37.9	.157
	Severe	8	27.6	2	6.9	
Neutrophil activity	None	16	55.2	20	69.0	**.010** *
	Mild	4	13.8	9	31.0	
	Moderate	7	24.1	0	0.0	
	Severe	2	6.9	0	0.0	
Metaplasia	Negative	27	93.1	25	86.2	.666
	Positive	2	6.9	4	13.8	
*H pylori*	Negative	15	51.7	20	69.0	**.002** **
	Mild positive	3	10.3	9	31.0	
	Moderate positive	5	17.2	0	0.0	
	Significant positivity	6	20.7	0	0.0	
Atrophy	Negative	27	93.1	26	89.7	1.000
	Positive	2	6.9	3	10.3	
Dysplasia	Negative	29	100.0	29	100.0	–
	Positive	0	0.0	0	0.0	–

Bold values indicates chi-square test (categorical data).

* *P* < .05.

** *P* < .01.

Comparisons of haematological parameters, including leukocytes, hemoglobin, hematocrit, mean corpuscular volume, platelet, lymphocyte, and neutrophil counts, showed no statistically significant differences between groups (*P* > .05). Biochemical evaluations encompassing glucose, urea, creatinine, alanine aminotransferase, aspartate aminotransferase, albumin, calcium, C-reactive protein, thyroid-stimulating hormone, and ferritin levels similarly revealed no significant differences between groups (*P* > .05). However, electrolyte assessments revealed that patients with bee allergy had significantly elevated sodium and potassium levels compared to the controls (*P* < .05). Table 3 shows the comparison of hemogram and biochemical parameters between the groups.

**Table 3:** Comparison of haemogram and biochemical parameters between the groups.

Laboratory findings	Bee allergy group (n: 29)	Control group (n: 29)	*P*
	Med.±S.D. Med. (Min.–Max.)	Med.±S.D. Med. (Min.–Max.)	
WBC†	7.17 ± 2.02 6.77 (3.05–11.78)	7.22 ± 2.23 7.01 (3.98–14.36)	.934
Hgb†	13.53 ± 1.99 13.4 (7.3–16.7)	13.39 ± 1.63 13.9 (10.6–17)	.774
Htc†	40.71 ± 5.2 41.3 (25.8–49.7)	40.56 ± 5.18 40.5 (31.4–49.9)	.912
MCV‡	87.29 ± 6.96 87.7 (59.4–100.3)	87.54 ± 7.31 87.9 (71.6–99.7)	.864
Plt†	243.03 ± 48.68 243 (167–342)	228.52 ± 35.96 224 (169–325)	.202
Lymphocyte†	2.09 ± 0.77 2.05 (0.08–3.44)	2.05 ± 0.44 2.05 (1.19–2.96)	.764
Neutrophil†	4.35 ± 1.57 4.08 (1.6–7.66)	4.47 ± 1.85 4.21 (1.47–8.96)	.788
Glucose‡	99.21 ± 18.68 96 (72–161)	95.14 ± 10.54 95 (79–127)	.432
Urea†	26.32 ± 8.11 25 (15–52)	30.07 ± 8.35 29 (14–49)	.091
Creatinine‡	0.8 ± 0.26 0.73 (0.53–1.7)	0.79 ± 0.15 0.81 (0.41–1.14)	.367
ALT‡	21.14 ± 13.8 16 (8–76)	18.66 ± 6.13 17 (10–34)	.821
AST‡	20.79 ± 8.02 19 (11–53)	18.45 ± 4.94 19 (10–29)	.279
Albumin‡	45.17 ± 4.22 46 (29.2–49.8)	46.3 ± 7.49 45.6 (37.8–75.6)	.967
Sodium‡	139.76 ± 3.15 140 (127–145)	138.66 ± 2.44 139 (133–142)	**.026** *
Potassium†	4.41 ± 0.46 4.5 (3.4–5.3)	4.16 ± 0.4 4 (3.5–4.9)	**.033** *
Calcium†	9.58 ± 0.39 9.6 (8.6–10.2)	9.78 ± 0.68 9.8 (8.3–10.9)	.182
CRP‡	5.83 ± 9.6 2.03 (0.16–40.52)	6.27 ± 11.48 2.7 (0.39–56.3)	.35
TSH‡	1.32 ± 0.88 1.21 (0.27–4.57)	1.29 ± 0.5 1.24 (0.39–2.1)	.586
Ferritin†	86.55 ± 98.71 48.61 (1.77–477.1)	115.42 ± 114.54 69.51 (17.69–494.34)	.181
PT†	10.21 ± 2.03 9.24 (7.48–15)	9.57 ± 1.11 9.19 (7.54–11.39)	.145
INR†	0.98 ± 0.09 0.97 (0.75–1.26)	0.98 ± 0.07 0.98 (0.84–1.1)	.787
APTT†	26.94 ± 2.66 26.9 (19.1–33.6)	27.21 ± 1.9 27.6 (23.1–31.02)	.656

Bold values indicates chi‐square test (categorical data).

ALT *=* alanine aminotransferase, APTT *=* activated partial thromboplastin time, AST *=* aspartate aminotransferase, CRP *=* C-reactive protein, Hct *=* hematocrit, Hgb *=* haemoglobin, INR = International Normalized Ratio, Max *=* maximum, MCV *=* mean corpuscular volüme, Med *=* median, Min *=* minimum, PT *=* prothrombin time, SD *=* standard deviation, TSH *=* thyroid-stimulating hormone, WBC *=* leucocytes.

† Independent sample *t* test.

‡ Mann–Whitney *U* test.

* *P* *<* .05.

Path analysis revealed a significant positive relationship between bee allergy and chronic inflammation (β = 0.41, *P* = .000), accounting for 16.8% of the variance (*R*² = 0.168). A significant positive association was also found between bee allergy and *H pylori* positivity (β = 0.38, *P* = .002), accounting for 14.3% of the variance (*R*² = 0.143). Furthermore, bee allergy demonstrated a statistically significant positive association with increased potassium levels (β = 0.28, *P* = .027, *R*² = 0.079). While sodium levels were positively associated with bee allergy, this association did not reach statistical significance (β = 0.20, *P* = .132). The results of the path analysis examining the effect of individuals’ bee allergy status on various parameters are shown in Table 4.

**Table 4:** The results of the path analysis examining the effect of individuals’ bee allergy status on various parameters.

Dependent variable	Path	Independent variable	B	SE	β (Beta)	*P*	*R* ^2^
Chronic inflammation	<---	Bee allergy	0.76	0.22	0.41	**.000** **	0.168
*H pylori*	<---	Bee allergy	0.76	0.25	0.38	**.002** **	0.143
Sodium	<---	Bee allergy	1.10	0.73	0.20	.132	0.038
Potassium	<---	Bee allergy	0.25	0.11	0.28	**.027** *	0.079

B: unstandardized path coefficient, β (Beta): standardized path coefficient.

*R*^2^: ratio of variance explained in dependent factors. Bold values indicates chi‐square test (categorical data).

* *P* < .05.

** *P* < .01.

**Figure 1.**
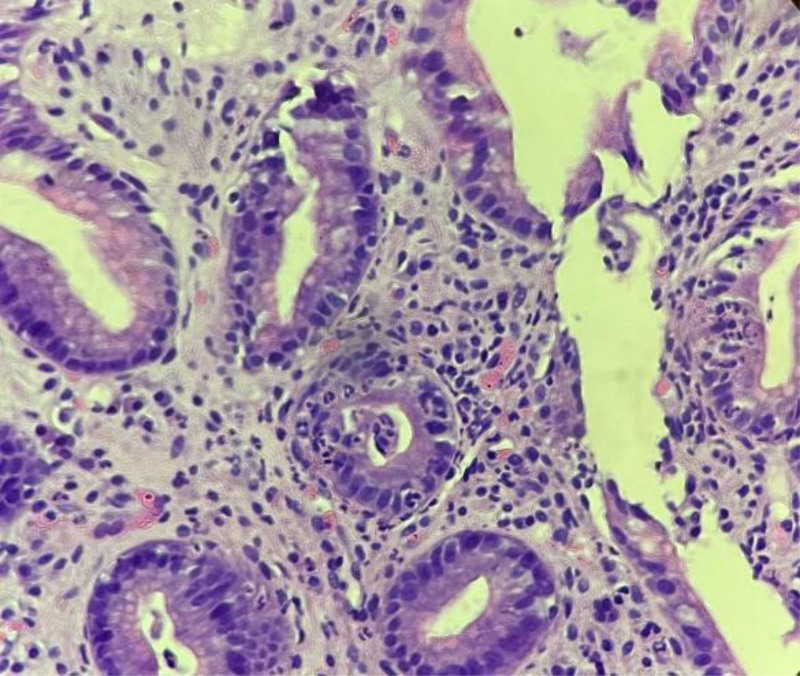
Gastric biopsy showing intraepithelial neutrophils (magnification 400×).

**Figure 2.**
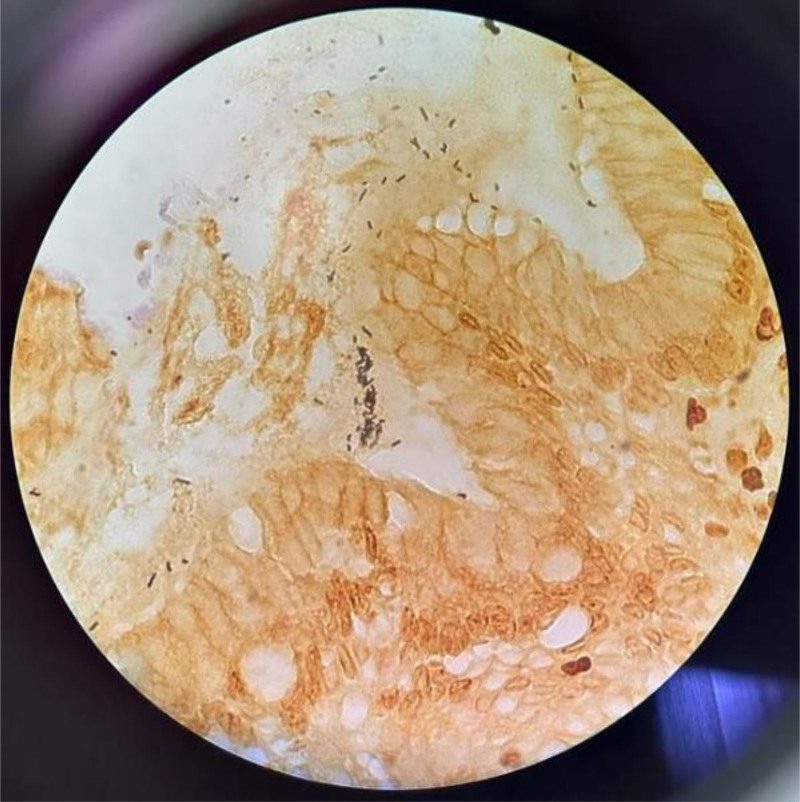
Gastric biopsy showing *Helicobacter pylori* (magnification 100×).

Figures 1 and 2 show histopathological examination of biopsies taken from the antrum mucosa of the stomach using forceps during endoscopy.

## 4. Discussion

This exploratory study, to the best of our knowledge, is the 1st to examine the potential association between bee allergy and alterations in gastric mucosal pathology and selected laboratory parameters. The findings of this study indicate that individuals with self-reported bee allergy exhibited higher rates of chronic gastric inflammation, increased neutrophil activity, and a greater prevalence of *H pylori* infection, along with elevated sodium and potassium levels, compared to matched controls.

The interaction between allergic conditions and gastrointestinal inflammation has become a growing focus in the field of immunological research. Research indicates that allergic responses, specifically those facilitated by immunoglobulin E, have the capacity to instigate systemic inflammatory cascades that impact numerous organ systems, including the gastrointestinal tract.^[8]^ The presence of increased chronic inflammation and neutrophil activity in the group of subjects diagnosed with bee allergies may reflect such systemic immunological influence. However, given the observational nature of the data, these associations should be interpreted with caution and not as direct evidence of a causal relationship.

The process of gastric colonization by *H pylori* is understood to be influenced by the mucosal immune environment of the host. Immune dysregulation associated with chronic allergic conditions has been demonstrated to alter epithelial barrier function or local inflammatory balance, thereby influencing microbial persistence.^[9,10]^ The higher frequency of *H pylori* positivity observed in the bee allergy group may be consistent with such a hypothesis, although the directionality and causation of this phenomenon remain to be established.

The observed electrolyte disturbances, specifically elevated potassium and sodium levels, may suggest broader systemic effects in individuals with bee allergy. As indicated by the preponderance of evidence, analogous findings have been reported in other allergic states, wherein mast cell activation and cytokine release have been demonstrated to exert an influence on cellular and metabolic processes.^[11,12]^ While these alterations in electrolyte parameters are noteworthy, it is essential to exercise caution in their interpretation, given the limitations imposed by the small sample size and the retrospective nature of the methodology employed.

The role of T helper 2 cytokines (e.g., interleukin 4, interleukin 5, and interleukin 13) in systemic inflammation and metabolic regulation has been increasingly recognized.^[13]^ These cytokines may exert an indirect influence on electrolyte transport and mucosal function, thereby providing a potential immunological context for the observed findings. However, given the exploratory nature of this study, these interpretations remain speculative and require further experimental validation.

This study provides preliminary evidence of a previously undiscovered correlation between bee allergy and gastric pathology. In view of the findings of the present study, it is clear that further detailed investigations are necessary to clarify the underlying immunological mechanisms, to confirm the clinical relevance and to develop potential preventive or therapeutic interventions. The findings of this study indicate that clinicians should consider conducting comprehensive evaluations of gastrointestinal health and electrolyte balance in individuals with a history of allergies, particularly those with a history of bee stings or other bee-related allergies.

### 4.1. Study limitations

The present study is subject to several limitations. Firstly, the retrospective design of the study limits the control that can be exercised over confounding factors, and reliance is placed on the accuracy of existing medical records. Secondly, the diagnosis of bee allergy was based on self-reported history rather than objective allergy testing, which may have introduced misclassification bias. The relatively modest sample size is a potential limitation, as it may restrict the generalizability of the findings, despite the presence of statistically significant results. Furthermore, although the groups were matched by age, gender, and symptoms, other unmeasured variables, such as environmental exposures or co-existing allergic conditions, were not controlled. Ultimately, the study did not include immunological or molecular analyses to explore underlying mechanisms. The findings of this study require further confirmation and expansion through the execution of future prospective studies. Such studies should involve larger cohorts and incorporate immunological profiling to achieve this objective.

## 5. Conclusion

The present study provides novel evidence suggesting that bee allergy may be associated with increased chronic gastric inflammation, heightened neutrophil activity, and higher *H pylori* positivity, as well as subtle alterations in electrolyte balance. This investigation represents the initial exploration of the relationships between systemic allergic responses and gastric mucosal pathology, and our findings suggest a potential immunological mechanism. While further research is needed to confirm these associations and clarify underlying mechanisms, this study establishes the foundation for a new perspective in allergy and gastroenterology, encouraging clinicians and researchers to consider the broader systemic effects of allergic conditions.

## Author contributions

**Formal analysis:** Berkan Acar, Ali Muhtaroğlu.

**Methodology:** Berkan Acar, Ali Muhtaroğlu.

**Writing – original draft:** Ali Muhtaroğlu, Kubilay İşsever.

**Writing – review & editing:** Kubilay İşsever, Ersin Kuloğlu, Sefer Aslan, Ahmet Cumhur Dülger.

## References

[1] Anvari S Miller J Yeh CY Davis CM. IgE-mediated food allergy. Clin Rev Allergy Immunol. 2019;57:244–60. 30370459 10.1007/s12016-018-8710-3

[2] Bégin P Waserman S Protudjer JLP Jeimy S Watson W. Immunoglobulin E (IgE)-mediated food allergy. Allergy Asthma Clin Immunol. 2024;20(Suppl 3):75. 39736801 10.1186/s13223-024-00930-7 PMC11684040

[3] Santos AF Riggioni C Du Toit G Skypala I. An algorithm for the diagnosis and management of IgE-mediated food allergy, 2024 update. Allergy. 2025;80:629–32. 39302341 10.1111/all.16321

[4] El-Seedi HR Refaey MS Abd El-Wahed AA. Bee products in the fight against *Helicobacter pylori* and molecular interactions. Microb Pathog. 2025;205:107707. 40378976 10.1016/j.micpath.2025.107707

[5] Nekoei S Rezvan M Khamesipour F Mayack C Molento MB Revainera PD. A systematic review of honey bee (Apis mellifera, Linnaeus, 1758) infections and available treatment options. Vet Med Sci. 2023;9:1848–60. 37335585 10.1002/vms3.1194 PMC10357250

[6] Daugule I Zavoronkova J Santare D. *Helicobacter pylori* and allergy: update of research. World J Methodol. 2015;5:203–11. 26713280 10.5662/wjm.v5.i4.203 PMC4686417

[7] Park AM Tsunoda I. *Helicobacter pylori* infection in the stomach induces neuroinflammation: the potential roles of bacterial outer membrane vesicles in an animal model of Alzheimer’s disease. Inflamm Regen. 2022;42:39. 36058998 10.1186/s41232-022-00224-8 PMC9442937

[8] Azouz NP Rothenberg ME. Mechanisms of gastrointestinal allergic disorders. J Clin Invest. 2019;129:1419–30. 30855279 10.1172/JCI124604 PMC6436864

[9] Zubeldia-Varela E Blanco-Pérez F Barker-Tejeda TC. The impact of high-IgE levels on metabolome and microbiome in experimental allergic enteritis. Allergy. 2024;79:3430–47. 38932655 10.1111/all.16202 PMC11657046

[10] Ma ZF Majid NA Yamaoka Y Lee YY. Food allergy and helicobacter pylori infection: a systematic review [published correction appears in Front Microbiol. 2016 Aug 08;7:1232. doi: 10.3389/fmicb.2016.01232.]. Front Microbiol. 2016;7:368. 27047479 10.3389/fmicb.2016.00368 PMC4804492

[11] Ness-Jensen E Langhammer A Hveem K Lu Y. *Helicobacter pylori* in relation to asthma and allergy modified by abdominal obesity: the HUNT study in Norway. World Allergy Organ J. 2019;12:100035. 31194177 10.1016/j.waojou.2019.100035 PMC6555905

[12] Arlt E Fraticelli M Tsvilovskyy V. TPC1 deficiency or blockade augments systemic anaphylaxis and mast cell activity. Proc Natl Acad Sci U S A. 2020;117:18068–78. 32661165 10.1073/pnas.1920122117 PMC7395440

[13] Lu HF Zhou YC Yang LT. Involvement and repair of epithelial barrier dysfunction in allergic diseases. Front Immunol. 2024;15:1348272. 38361946 10.3389/fimmu.2024.1348272 PMC10867171

